# Comparison of skin biopsy sample processing and storage methods on high dimensional immune gene expression using the Nanostring nCounter system

**DOI:** 10.1186/s13000-020-00974-4

**Published:** 2020-05-15

**Authors:** Jelena Vider, Andrew Croaker, Amanda J. Cox, Emma Raymond, Rebecca Rogers, Stuart Adamson, Michael Doyle, Blake O’Brien, Allan W. Cripps, Nicholas P. West

**Affiliations:** 1 School of Medical Science and Menzies Health Institute QLD, Griffith University, Gold Coast, Queensland 4222 Australia; 2 Systems Biology and Data Science, Menzies Health Institute QLD, Griffith University, Gold Coast, Queensland 4222 Australia; 3 School of Health and Human Sciences, Southern Cross University, Lismore, NSW Australia; 4 Toormina Medical Centre, Toormina, NSW Australia; 5 Wesley Medical Research, Auchenflower, QLD Australia; 6 Brain Cancer Biobanking Australia, Camperdown, NSW Australia; 7 Mid-West Aero Medical, Geraldton, Perth Australia; 8 Sullivan &Nicolaides Pathology, Bowen Hills, QLD Australia; 9 School of Medicine and Menzies Health Institute QLD, Griffith University, Gold Coast, QLD Australia

**Keywords:** Skin biopsy, Immune gene expression, PanCancer immune profiling panel, Nanostring, Sample processing

## Abstract

**Background:**

Digital multiplex gene expression profiling is overcoming the limitations of many tissue-processing and RNA extraction techniques for the reproducible and quantitative molecular classification of disease. We assessed the effect of different skin biopsy collection/storage conditions on mRNA quality and quantity and the NanoString nCounter™ System’s ability to reproducibly quantify the expression of 730 immune genes from skin biopsies.

**Methods:**

Healthy human skin punch biopsies (*n* = 6) obtained from skin sections from four patients undergoing routine abdominoplasty were subject to one of several collection/storage protocols, including: i) snap freezing in liquid nitrogen and transportation on dry ice; ii) RNAlater (ThermoFisher) for 24 h at room temperature then stored at − 80 °C; iii) formalin fixation with further processing for FFPE blocks; iv) DNA/RNA shield (Zymo) stored and shipped at room temperature; v) placed in TRIzol then stored at − 80 °C; vi) saline without RNAse for 24 h at room temperature then stored at − 80 °C. RNA yield and integrity was assessed following extraction via NanoDrop, QuantiFluor with RNA specific dye and a Bioanalyser (LabChip24, PerkinElmer). Immune gene expression was analysed using the NanoString Pancancer Immune Profiling Panel containing 730 genes.

**Results:**

Except for saline, all protocols yielded total RNA in quantities/qualities that could be analysed by NanoString nCounter technology, although the quality of the extracted RNA varied widely. Mean RNA integrity was highest from samples that were placed in RNALater (RQS 8.2 ± 1.15), with integrity lowest from the saline stored sample (RQS < 2). There was a high degree of reproducibility in the expression of immune genes between all samples with the exception of saline, with the number of detected genes at counts < 100, between 100 and 1000 and > 10,000 similar across extraction protocols.

**Conclusions:**

A variety of processing methods can be used for digital immune gene expression profiling in mRNA extracted from skin that are comparable to snap frozen skin specimens, providing skin cancer clinicians greater opportunity to supply skin specimens to tissue banks. NanoString nCounter technology can determine gene expression in skin biopsy specimens with a high degree of sensitivity despite lower RNA yields and processing methods that may generate poorer quality RNA. The increased sensitivity of digital gene expression profiling continues to expand molecular pathology profiling of disease.

## Introduction

Molecular profiling of tissue for insight into mechanisms of disease, stratification of individuals for disease risk and to monitor therapeutic responses is rapidly increasing due to advances in technology. Driven by high-throughput molecular technology, such as digital sequencing, there is a growing body of molecular biomarker data across cancer phenotypes that aim to allow for personalised medical approaches that minimise unnecessary treatment.

A key consideration in molecular biomarker analysis is the need to extract high quality RNA from tissue samples [1]. The cross-linking of nucleic acids to proteins and other cellular components, such as in formalin fixation, makes the extraction of high-quality RNA difficult [2] . In recent years, the development of the NanoString nCounter platform, which utilises direct, digital quantitation of mRNA transcripts via hybridisation to colour-coded sequence specific probes, has overcome the limitations associated with detecting nucleic acid targets at all levels of biological expression [3]. The ability to multiplex targets reproducibly from RNA extracted from formalin fixed paraffin embedded (FFPE) samples has provided greater avenues for molecular research, particularly for clinicians located at sites not located near pathology or research facilities.

Various methods are also available for RNA protection, such as with TRIzol [4] or RNAlater, to overcome challenges with low quantity or low quality mRNA derived from FFPE samples. Given that mRNA quality and concentration impacts data quality, it is necessary to optimise collection/storage techniques for the sample processing [5]. Reliable and reproducible methods of obtaining sufficient amounts of high-quality RNA from tissue remain a challenge for biomarker studies, in particular studies involving skin samples. Skin biopsies are recognised to be difficult samples to achieve consistently high-quality RNA [6]. Investigations with the nCounter technology indicate the ability to measure mRNA with low yield and sub-optimal RNA quality. In this study we compared the impact of six tissue-processing methods on skin biopsies total RNA yield/integrity and the multiplex gene expression using the NanoString nCounter analysis system.

## Methods

This was a comparison of immune gene expression from six skin tissue biopsy RNA extraction methods collected from three healthy patients undergoing abdominoplasty, with biopsies 3 and 4 collected from the same patient. All six methods were performed on abdominoplasty tissue collected from each person. Following excision of tissue, six 4 mm biopsies were collected with standard techniques. The study was conducted under approval from the Griffith University Human Research Ethics Committee and the United HealthCare Human Research Ethics Committee (HMR/05/15/HREC).

### Tissue processing and storage

Following collection of the six skin biopsies from tissue from each patient the following storage and transport procedures were used: i) snap freezing in liquid nitrogen and transportation on dry ice; ii) RNAlater (ThermoFisher Scientific, Waltham, MA, USA) for 24 h at room temperature then stored at − 80 °C; iii) formalin fixation and storage of FFPE blocks at room temperature; iv) DNA/RNA Shield (Zymo, Irvine, CA, USA) stored and shipped at room temperature; v) placed in TRIzol (ThermoFisher Scientific, Waltham, MA, USA) then stored at − 80 °C; vi) 0.15 ml saline without RNAse for 24 h at room temperature then stored at − 80 °C. First homogenization of skin biopsies using ZR BashingBead Lysis Tubes (Zymo) and Tissue Lyser II (Qiagen) was unsuccessful, therefore it was re-done using gentleMACS octo and M tubes (Miltenyi Biotec). For the samples processed with liquid nitrogen, saline and RNAlater, RNA was extracted using the Maxwell® RSC simplyRNA Tissue Kit (Promega, Madison, USA). For the FFPE samples the RNeasy® mini kit (QIAGEN, Hilden, Germany) and ReliaPrep™ FFPE Total RNA Miniprep System (data is not shown) were used for RNA extraction. From samples in TRIzol RNA was extracted using the Direct-Zol™ RNA kit (Zymo, Irvine, CA, USA) while the Quick RNA™ Miniprep Kit (Zymo, Irvine, CA, USA) was used for extraction of RNA from DNA/RNA shield (Zymo, Irvine, CA, USA) stored biopsies. After isolation RNA samples were aliquoted and stored at − 80 °C until further analysis.

### RNA yield and integrity

RNA extraction was performed in an RNAse-free environment following the manufacturer’s protocol for each kit. The concentration of extracted RNA (ng/μL) was assessed using three different methods: i) UV-spectrophotometry (NanoDrop, ThermoScientific); ii) LabChip24 with Standard and Pico sensitivity RNA reagents (PerkinElmer); iii) Quantifluor direct RNA dye (Promega). A_260 /_ A_280_ ratio was measured with the NanoDrop 1000 UV-Vis spectrophotometer (ThermoScientific, Massachusettes, United States) with an A_260 /_ A_280_ ratio > 1.9 considered an indicator of pure RNA. RNA quality score (RQS) was calculated by a LabChip 24 bioanalyzer (PerkinElmer). Based on data using RNA Pico Sensitivity Reagent Kit, all RNA samples except LN1, LN2, RL1 and RL2 were concentrated using the Zymo RNA Concentrator kit (Zymo). After concentration, RNA was assessed using Quantifluor direct RNA dye (Promega) and LabChip 24 RNA Pico Sensitivity Reagent Kit (PerkinElmer).

### NanoString gene expression analysis

Immune gene expression analysis was undertaken using the NanoString nCounter analysis system (NanoString Technologies, Seattle, WA) using the commercially available nCounter PanCancer Immune Profiling panel kit. The PanCancer Immune profiling panel contains *n* = 730 genes of key inflammatory pathways and *n* = 40 reference/housekeeping genes. The manufacturer’s protocol was followed with small modification in that 300 ng of total RNA extracted from skin biopsies was hybridised with probes at 65 °C for 24 h. Samples were processed on the NanoString Prep Station and the target-probe complex was immobilised onto the analysis cartridge. Cartridges were scanned by the nCounter Digital Analyser for digital counting of molecular barcodes corresponding to each target at 280 fields of view.

### Data approach

Gene expression data was analysed using the Advanced Analysis Module in the nSolver™ Analysis Software version 4.0 from NanoString Technologies (NanoString Technologies, WA, USA) and TIGR Multi-Experiment Viewer (http://mev.tm4.org). The Advanced Analysis Module enables quality control (QC), normalisation, cluster analysis, differential gene expression (DGE), Pathview Plots and immune cell profiling. Raw data was normalised by subtracting the mean plus one standard deviation of eight negative controls while technical variation was normalised through internal positive controls. Data was corrected for input volume via internal housekeeping genes using the geNorm algorithm. Immune cell scores were determined using cell specific gene expression from The Cancer Genome Atlas (TCGA) as detailed in [7, 8]. A Pearson correlation was used to determine degree of similarity of gene expression counts with significance accepted at *p* < 0.001.

## Results

### Yield and integrity of extracted RNA

The average concentrations of extracted RNA for each processing method is shown in Table 1. RNA could be extracted from all samples, although the concentration and quality varied widely between and within processing methods. We found that in samples from one patient (set 3 and 4) stored in liquid nitrogen, RNAlater and saline RNA extraction did not yield enough RNA for nCounter Nanostring assay. RNA extracted from FFPE samples exhibited the most consistent concentrations and RQ scores while RNA / DNA shield resulted in consistent RQ scores but variable concentrations. RNA yield from biopsies stored in Liquid nitrogen, RNAlater and TRIzol of same participant was very low (set 3 and 4). We considered RNA concentration data assessed by UV-spectrophotometry (NanoDrop 1000, ThermoScientific) as unreliable for use with nCounter Nanostring system.

**Table 1:** RNA concentration and quality scores in the collected samples

Method	Concentration	A260 / A280	Concentration	RQS	nCounter
	NanoDrop (ng/μL)		Quantifluor (ng/μL)		counts
LN1	315.48	1.9	67.2	6.5	ok
LN2	106.69	1.83	86.4	4.8	ok
LN3	19.44	2.47	0.2	NA	NA
LN4	90.22	1.52	1.7	NA	ND
RL1	88.83	2.11	69.5	9.4	ok
RL2	68.38	2.06	40.2	8.1	ok
RL3	15.6	2.44	1.6	7.1	ND
RL4	9.01	3.3	ND	ND	NA
Q_FFPE1	68.02	1.63	13	5.3	ok
Q_FFPE2	63.62	1.74	25.6	5.4	ok
Q_FFPE3	30.13	1.94	16	5.6	ok
Q_FFPE4	36.21	1.82	13.2	5.9	ok
P-FFPE1	24.44	1.89	11.0	4.5	ok
P-FFPE2	49.53	1.79	19.0	4.4	ok
P-FFPE3	48.61	1.98	35.9	4	ok
P-FFPE4	45.24	1.87	20.8	3.2	NA
TR1	0.79	1.5	49.4	4.6	ok
TR2	1.91	2.55	37.9	4.6	ok
TR3	0.1	0.21	12	NA	ok
TR4	0	0.68	16.7	4.9	ok
RS1	20.84	2.88	34.6	4.2	ok
RS2	9.12	3.32	11	5.5	ok
RS3	3.65	−3.43	4.5	6.2	ok
RS4	19.34	2.49	4.8	5.3	NA
S1	127.41	1.59	1.8	ND	ND
S2	21.12	2.23	3.5	2.1	ND
S3	11.87	1.76	ND	ND	ND
S4	93.72	1.53	ND	ND	NA

Processing and storage methods: LN- liquid nitrogen; RL – RNAlater; FFPE – formalin fixed paraffin embedded; TR – trizol; RS – RNA/DNA shield; S – saline; ng – nanograms; μL – microlitres; RQS – RNA integrity number. ND - under limit of detection; NA – sample was not run on nCounter. Quantifluor and RQS data was acquired after RNA concentration with.

Zymo RNA Concentrator kit (Zymo).

### Immune gene expression

**Fig. 1:**
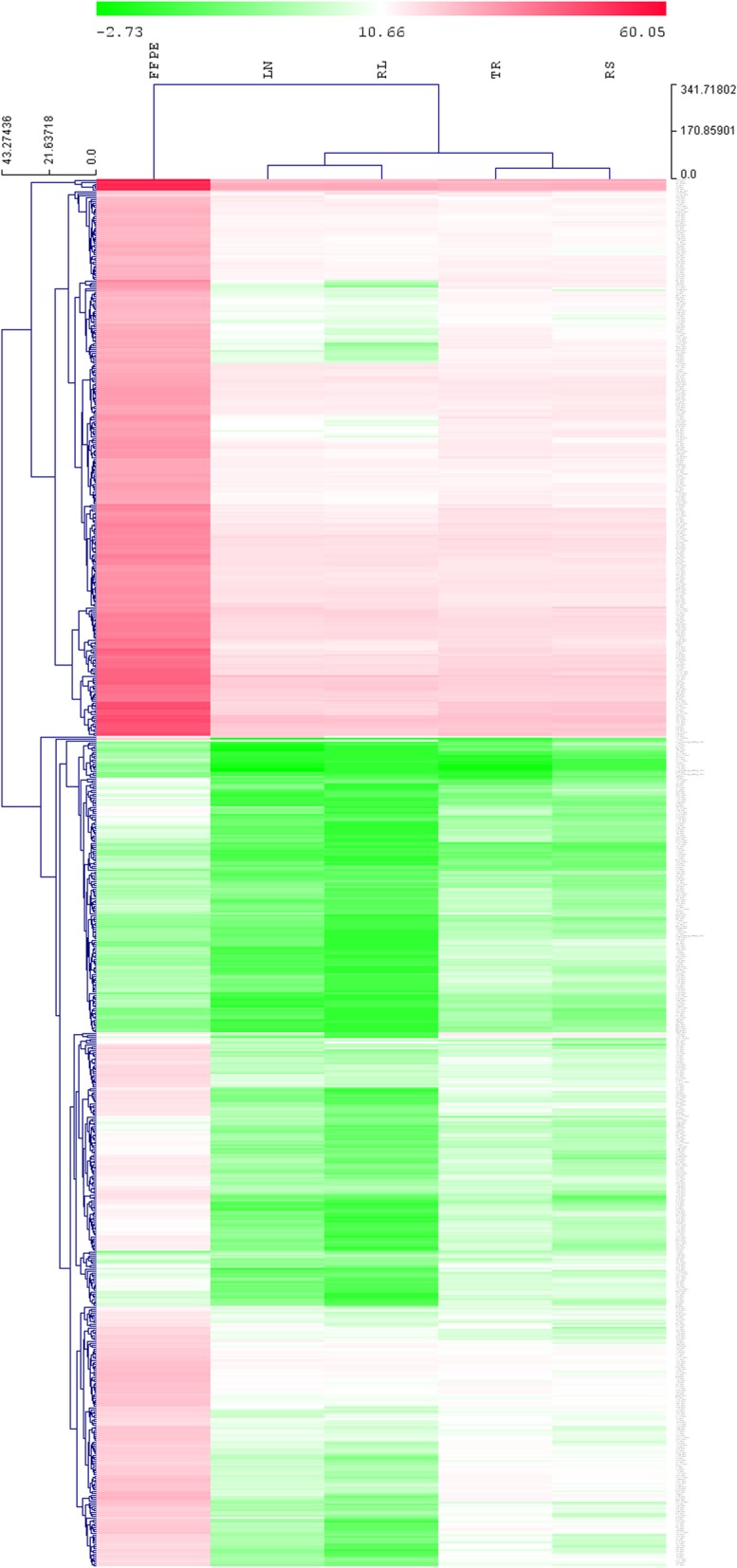
A hierarchical cluster heatmap of the 730 immune genes by group. With the exception of FFPE which shows higher immune gene expression, the groups show similar gene expression counts. Each row is a gene and each column a group. Green is low expression and red is high expression. Immune gene expression from samples stored in saline are not included. LN- liquid nitrogen; RL – RNAlater; FFPE – formalin fixed paraffin embedded; TR – trizol; RS – RNA / DNA shield

Counts for genes above background threshold, below 100, between 101 and 1000 and above 1000 by sample are shown in Table 2. Total RNA extracted from FFPE, LN and RNAlater returned the highest gene expression counts above background threshold levels (the geometric mean of the negative control samples). All samples showed similar counts at expression levels > 1000. The similarity across samples is depicted in Fig. 1, which is a heatmap from an unsupervised clustering of the 730 genes included in the PanCancer Immune Profiling panel. On average the FFPE samples had higher gene expression counts than total RNA extracted from samples using other protocols. There was a high correlation co-efficient in immune gene expression counts between the tissue processing and RNA extraction methods (r = ~ 0.88–0.97; *p* < 0.001).

**Table 2:** Immune gene expression counts above background, below 100, between 100 and 1000 and above 1000 by mRNA extraction method. Values presented are mean counts ± standard deviation of the four processing storage methods. No counts could be determined from the saline samples

	FFPE	LN	TR	RS	RLT
Above background	730	663.5 ± 53.03	719.67 ± 8.96	730	579.50 ± 65.76
Below 100	494 ± 8.28	523 ± 25.45	504 ± 13.45	514.75 ± 4.5	547.5 ± 9.19
between 100 and 1000	199.25 ± 9.46	174.5 ± 17.67	193.33 ± 14.01	180.5 ± 16.7	155 ± 9.89
above 1000	36.75 ± 1.5	32.5 ± 7.77	32.66 ± 0.57	34.75 ± 4.34	27.5 ± 0.7

Processing and storage methods: LN- liquid nitrogen; RL – RNAlater; FFPE – formalin fixed paraffin embedded; TR – trizol; RS – RNA / DNA shield;

## Discussion

The role of molecular profiling in pathology to classify disease was recognised in 2014 through the formalisation of an informatics subdivision within the Association for Molecular Pathology given the growing use of high throughput quantitative data to deliver health care [9]. A recognised limitation to the generation of high-quality omics data is RNA yield and quality [6]. This study compared total RNA yield and quality on immune gene expression from healthy skin biopsies across six tissue processing/storage protocols. All protocols yielded RNA quantities with wide ranges of quality and concentration metrics. Skin tissue is recognised to be difficult to reliably extract high quality mRNA as a result of sub-optimal biopsy procedures not yielding sufficient quantity of tissue, RNase activity and the nature of the collagen matrix [6]. Recent studies highlight the difficulties of obtaining sufficient RNA from skin even with the latest extraction techniques [6]. In our investigation, formalin fixation and storage in RNA / DNA shield yielded the most consistent quality scores across all samples. Importantly, all processing methods except saline storage (RNA degradation) were compatible with NanoString nCounter analysis, highlighting the versatility of this hybridisation-based application to overcome the limitations of extraction protocols for undertaking molecular profiling. This versatility provides researchers and pathologists with simpler options to collect and store biological samples for more comprehensive classification of disease.

Variation in RNA quality results in inaccurate and misleading changes in molecular profiling, underpinning the need for reliable and reproducible protocols for the processing of tissue and extraction of RNA [10]. Numerous studies have compared extraction kits for the isolation of nucleic acids from FFPE tissue, with key factors to consider listed for researchers prior to undertaking experimental processes [1, 11]. While DNA/RNA Shield yielded similar quality scores to RNA extracted from FFPE tissue, there was substantial variation in the total yield of RNA. The highest quality RNA was obtained from the samples stored in RNAlater, although there was a high degree of variation in the quality scores and RNA concentration from samples utilising this protocol. Overall, FFPE samples appear to provide the most consistent RNA quality scores and yields.

The NanoString nCounter Analysis system has been one of the latest advances in genomic technology for molecular profiling. As a hybridisation-based system, the technology eliminates the need for amplification bias common to PCR for direct counting of molecular transcripts. Research has demonstrated that the NanoString System is able to quantify transcripts from total RNA of lower quality and quantity, potentially providing researchers with additional options for the collection of tissue for molecular profiling. We utilised the PanCancer Immune Profiling kit to undertake broad-based molecular profiling of mRNA extracted from tissue using the various tissue processing techniques. The technology had high sensitivity of target detection across the sample set even at lower quality scores and yields, which is consistent with previous research [3, 12]. Absolute gene expression counts were similar across the various skin tissue processing and RNA extraction protocols. Our data highlight the utility of the system for use with a range of tissue processing and RNA extraction protocols. This gives primary care physicians, researchers and pathologists, particularly in locations without access to liquid nitrogen facilities, greater flexibility to collect skin samples for the molecular classification of disease, particularly in oncology, aging, the endotypes of atopic dermatitis and other hypersensitivity reactions [13]. Provided consistency in the use of these methods by protocol, this gives researchers and primary care skin clinicians a wide variety of options to undertake molecular profiling of biological samples.

In conclusion, our study shows that several tissue processing and extraction techniques successfully isolate RNA for analysis using high throughput digital counting. We observed substantial variation in the quality and yield of these techniques, with tissue stored in FFPE blocks providing the most consistent yield and quality scores in all participants. We note a number of limitations, in particular the small number of samples per protocol, that each processing method utilised a different RNA extraction method, that the results relate to skin samples only and that these samples were fresh tissue not older samples so caution should be taken in extrapolating these results. Many of these limitations are consistent with clinical research and increases the ecological validity of the results for research and pathology purposes. Despite the variation and quality of mRNA, the NanoString nCounter analysis system was able to quantify 730 genes across protocols with a high degree of similarity, highlighting the benefits of hybridisation-based technology for molecular profiling.

## Data Availability

All data is available from the corresponding author.

## Abbreviations

FFPE: Formalin fixed paraffin embedded
HREC: Human Research Ethics Committee
DGE: Differential gene expression
TCGA: The Cancer Genome Atlas
LN: Liquid nitrogen
RL: RNAlater
TR: Trizol
RNA/DNA Shield: RNA / DNA shield
ng: Nanograms
μl: Microlitres
RQS: RNA integrity number
